# 10-Phenyl-6b,7,8,9,9a,10-hexa­hydro-6*H*-cyclo­penta­[4,5]pyrano[3,2-*c*]chromen-6,9-dione

**DOI:** 10.1107/S1600536811006192

**Published:** 2011-03-12

**Authors:** Zhen Qiao, Li Liu, Dong Wang

**Affiliations:** a Beijing National Laboratory for Molecular Science (BNLMS), CAS Key Laboratory for Molecular Recognition and Function, Institute of Chemistry, Chinese Academy of Sciences, Beijing 100190, People’s Republic of China

## Abstract

In the title compound, C_21_H_16_O_4_, the dihedral angle between the phenyl ring and the 2*H*-chromene ring system is 59.8 (2)°. The crystal packing is stabilized by weak π–π stacking inter­actions [centroid–centroid distances = 3.667 (2) Å] and inter­molecular C—H⋯O hydrogen-bonding inter­actions.

## Related literature

For applications of coumarin, see: Vu *et al.* (2008 ▶); Maresca *et al.* (2009 ▶); Maresca *et al.* (2010 ▶). For bond-length data, see: Allen *et al.* (1987 ▶).
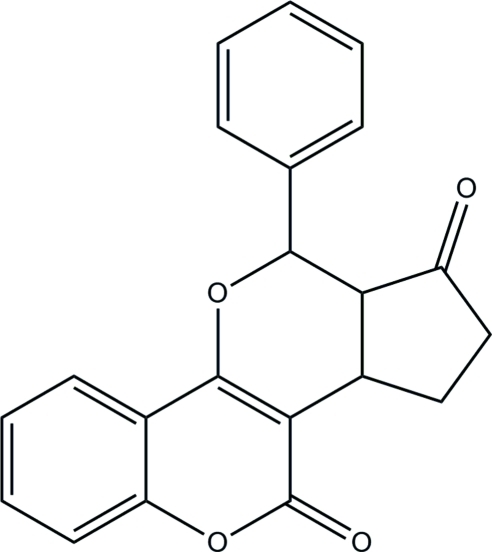


## Experimental

### 

#### Crystal data

C_21_H_16_O_4_
*M* *_r_* = 332.34
Monoclinic, 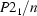

*a* = 9.1672 (14) Å
*b* = 8.6538 (14) Å
*c* = 19.899 (3) Å
β = 91.295 (3)°
*V* = 1578.2 (4) Å^3^
*Z* = 4
Mo *K*α radiation
μ = 0.10 mm^−1^
*T* = 173 K
0.50 × 0.50 × 0.41 mm

#### Data collection

Rigaku Saturn724+ CCD diffractometer
Absorption correction: numerical (*CrystalClear*; Rigaku, 2007 ▶) *T*_min_ = 0.953, *T*_max_ = 0.961
13128 measured reflections
3608 independent reflections
3469 reflections with *I* > 2σ(*I*)
*R*_int_ = 0.030

#### Refinement

*R*[*F* ^2^ > 2σ(*F* ^2^)] = 0.055
*wR*(*F* ^2^) = 0.144
*S* = 1.10
3608 reflections
226 parameters
H-atom parameters constrained
Δρ_max_ = 0.38 e Å^−3^
Δρ_min_ = −0.22 e Å^−3^

### 

Data collection: *CrystalClear* (Rigaku, 2007 ▶); cell refinement: *CrystalClear*; data reduction: *CrystalClear*; program(s) used to solve structure: *SHELXS97* (Sheldrick, 2008 ▶); program(s) used to refine structure: *SHELXL97* (Sheldrick, 2008 ▶); molecular graphics: *SHELXTL* (Sheldrick, 2008 ▶); software used to prepare material for publication: *SHELXTL* (Sheldrick, 2008 ▶).

## Supplementary Material

Crystal structure: contains datablocks I, global. DOI: 10.1107/S1600536811006192/hg2800sup1.cif

Structure factors: contains datablocks I. DOI: 10.1107/S1600536811006192/hg2800Isup2.hkl

Additional supplementary materials:  crystallographic information; 3D view; checkCIF report

## Figures and Tables

**Table 1:** Hydrogen-bond geometry (Å, °)

*D*—H⋯*A*	*D*—H	H⋯*A*	*D*⋯*A*	*D*—H⋯*A*
C8—H8*A*⋯O3^i^	1.00	2.45	3.4042 (19)	160
C17—H17*A*⋯O2^ii^	0.95	2.54	3.322 (2)	140

Symmetry codes: (i) 
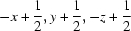
; (ii) 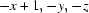.

**Fig. 1.**
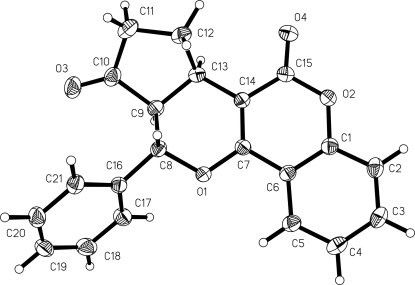
The molecular structure of (I), with atom labels and 40% probability displacement ellipsoids for non-H atoms.

## supplementary crystallographic information

### Comment

Coumarins constitute a ubiquitous class of heterocycles found in numerous natural products, food industry, marketed drugs, and drug candidates [Vu *et al.*, 2008; Maresca *et al.*, 2009; Maresca *et al.*, 2010]. Alkylations of electron-rich arenes such as 4-hydroxycoumarin are of great importance for the synthesis of many natural products and pharmaceuticals. Therefore, multiple approaches have been undertaken to develop catalytic enantioselective additions of 4-hydroxycoumarin to α,β-unsaturated carbonyl compounds. In this context the use of cyclic Morita Baylis Hillman alcohol is of particular interest since they not only exhibit regioselectivity but also can be cyclized readily followed by reaction of the resultant allylic cation with a suitable O nucleophile. In continuation of our work in this direction, we report here the crystal structure of the title compound.

In title compound, all bond lengths in the molecular are normal (Allen *et al.*, 1987). The dihedral angle between benzene (C16—C21) and 2*H*-chromene (C1—C7/C14/C15/O1) rings is 59.8 (2) °. π—π interactions are indicated by the short distance (*Cg*1···*Cg*2 distance of 3.667 (2) Å, symmetry code: 1 - *x*,1 - *y*,*z*) between the centroids of the 2*H*-pyran ring (C1/C6/C7/C14/C15/O2) (*Cg*1) and benzene ring C1—C6 (*Cg*2) (Table 1). There are weaker C—H···O intermolecular interactions, which stabilized the structure (Table 1).

### Experimental

A mixture of 9-amino-9-deoxyepiquinine QA (20 mol %) in the combination with TFA (40 mol %) exhibited high catalytic activity for the Michael addition followed by cycloaddition of 4-hydroxycoumarin to cyclopent-2-enone-derived MBH alcohol in acetone at 60 °C for 72 h, yield 61%. Single crystals suitable for X-ray measurements were obtained by recrystallization from acetonitrile at room temperature.

### Refinement

H atoms were positioned geometrically and refined using a riding model, with C—H = 0.95 to 1.00 Å and with *U*_iso_(H) = 1.2 times *U*_eq_(C).

### Crystal data

**Table:**

C_21_H_16_O_4_	*F*(000) = 696
*M**_r_* = 332.34	*D*_x_ = 1.399 Mg m^−^^3^
Monoclinic, *P*2_1_/*n*	Mo *K*α radiation, λ = 0.71073 Å
Hall symbol: -P 2yn	Cell parameters from 4768 reflections
*a* = 9.1672 (14) Å	θ = 1.0–27.5°
*b* = 8.6538 (14) Å	µ = 0.10 mm^−^^1^
*c* = 19.899 (3) Å	*T* = 173 K
β = 91.295 (3)°	Block, colorless
*V* = 1578.2 (4) Å^3^	0.50 × 0.50 × 0.41 mm
*Z* = 4	

### Data collection

**Table:**

Rigaku Saturn724+ CCD diffractometer	3608 independent reflections
Radiation source: sealed tube	3469 reflections with *I* > 2σ(*I*)
graphite	*R*_int_ = 0.030
ω scans at fixed χ = 45°	θ_max_ = 27.5°, θ_min_ = 3.1°
Absorption correction: numerical (*CrystalClear*; Rigaku, 2007)	*h* = −11→11
*T*_min_ = 0.953, *T*_max_ = 0.961	*k* = −10→11
13128 measured reflections	*l* = −22→25

### Refinement

**Table:**

Refinement on *F*^2^	Primary atom site location: structure-invariant direct methods
Least-squares matrix: full	Secondary atom site location: difference Fourier map
*R*[*F*^2^ > 2σ(*F*^2^)] = 0.055	Hydrogen site location: inferred from neighbouring sites
*wR*(*F*^2^) = 0.144	H-atom parameters constrained
*S* = 1.10	*w* = 1/[σ^2^(*F*_o_^2^) + (0.0704*P*)^2^ + 0.6329*P*] where *P* = (*F*_o_^2^ + 2*F*_c_^2^)/3
3608 reflections	(Δ/σ)_max_ < 0.001
226 parameters	Δρ_max_ = 0.38 e Å^−^^3^
0 restraints	Δρ_min_ = −0.22 e Å^−^^3^

### Special details

**Table:**

Geometry. All e.s.d.'s (except the e.s.d. in the dihedral angle between two l.s. planes) are estimated using the full covariance matrix. The cell e.s.d.'s are taken into account individually in the estimation of e.s.d.'s in distances, angles and torsion angles; correlations between e.s.d.'s in cell parameters are only used when they are defined by crystal symmetry. An approximate (isotropic) treatment of cell e.s.d.'s is used for estimating e.s.d.'s involving l.s. planes.
Refinement. Refinement of *F*^2^ against ALL reflections. The weighted *R*-factor *wR* and goodness of fit *S* are based on *F*^2^, conventional *R*-factors *R* are based on *F*, with *F* set to zero for negative *F*^2^. The threshold expression of *F*^2^ > σ(*F*^2^) is used only for calculating *R*-factors(gt) *etc*. and is not relevant to the choice of reflections for refinement. *R*-factors based on *F*^2^ are statistically about twice as large as those based on *F*, and *R*- factors based on ALL data will be even larger.

### Fractional atomic coordinates and isotropic or equivalent isotropic displacement parameters (Å^2^)

**Table:**

	*x*	*y*	*z*	*U*_iso_*/*U*_eq_	
O1	0.50827 (10)	0.14483 (13)	0.10143 (5)	0.0292 (2)	
O2	0.30494 (11)	0.31578 (13)	−0.06936 (5)	0.0342 (3)	
O3	0.21059 (14)	−0.10952 (16)	0.24113 (6)	0.0472 (3)	
O4	0.09368 (13)	0.21341 (18)	−0.04206 (7)	0.0509 (4)	
C1	0.45295 (15)	0.33318 (17)	−0.05937 (7)	0.0293 (3)	
C2	0.52778 (18)	0.41320 (18)	−0.10830 (8)	0.0344 (3)	
H2A	0.4773	0.4543	−0.1465	0.041*	
C3	0.67665 (18)	0.43206 (18)	−0.10057 (8)	0.0358 (3)	
H3A	0.7289	0.4869	−0.1337	0.043*	
C4	0.75115 (17)	0.37144 (19)	−0.04468 (8)	0.0358 (3)	
H4A	0.8538	0.3846	−0.0400	0.043*	
C5	0.67574 (16)	0.29218 (18)	0.00397 (8)	0.0321 (3)	
H5A	0.7267	0.2512	0.0420	0.039*	
C6	0.52458 (15)	0.27221 (16)	−0.00277 (7)	0.0268 (3)	
C7	0.43568 (15)	0.19240 (16)	0.04545 (7)	0.0260 (3)	
C8	0.41941 (15)	0.10574 (17)	0.15850 (7)	0.0275 (3)	
H8A	0.3765	0.2025	0.1771	0.033*	
C9	0.29535 (15)	−0.00278 (17)	0.13564 (7)	0.0289 (3)	
H9A	0.3352	−0.1037	0.1198	0.035*	
C10	0.18782 (18)	−0.02793 (19)	0.19294 (8)	0.0356 (3)	
C11	0.0505 (2)	0.0604 (3)	0.17786 (11)	0.0564 (5)	
H11A	0.0224	0.1227	0.2173	0.068*	
H11B	−0.0305	−0.0110	0.1661	0.068*	
C12	0.08425 (18)	0.1647 (2)	0.11868 (9)	0.0412 (4)	
H12A	0.1231	0.2656	0.1342	0.049*	
H12B	−0.0039	0.1824	0.0900	0.049*	
C13	0.20060 (15)	0.07317 (18)	0.08039 (7)	0.0308 (3)	
H13A	0.1506	−0.0100	0.0537	0.037*	
C14	0.29132 (15)	0.16801 (17)	0.03381 (7)	0.0285 (3)	
C15	0.22106 (16)	0.22987 (19)	−0.02619 (8)	0.0343 (3)	
C16	0.51992 (15)	0.03462 (17)	0.21074 (7)	0.0274 (3)	
C17	0.62801 (17)	−0.06787 (19)	0.19234 (8)	0.0360 (3)	
H17A	0.6423	−0.0896	0.1462	0.043*	
C18	0.71543 (18)	−0.1389 (2)	0.24094 (9)	0.0440 (4)	
H18A	0.7886	−0.2101	0.2280	0.053*	
C19	0.69637 (19)	−0.1066 (2)	0.30784 (9)	0.0453 (4)	
H19A	0.7564	−0.1553	0.3411	0.054*	
C20	0.59009 (19)	−0.0033 (2)	0.32673 (8)	0.0413 (4)	
H20A	0.5775	0.0194	0.3729	0.050*	
C21	0.50157 (17)	0.06724 (18)	0.27822 (7)	0.0325 (3)	
H21A	0.4283	0.1380	0.2913	0.039*	

### Atomic displacement parameters (Å^2^)

**Table:**

	*U*^11^	*U*^22^	*U*^33^	*U*^12^	*U*^13^	*U*^23^
O1	0.0241 (5)	0.0389 (6)	0.0247 (5)	−0.0026 (4)	0.0003 (4)	0.0033 (4)
O2	0.0283 (5)	0.0438 (6)	0.0306 (5)	0.0048 (4)	0.0013 (4)	0.0074 (4)
O3	0.0493 (7)	0.0534 (8)	0.0387 (6)	−0.0192 (6)	0.0001 (5)	0.0139 (5)
O4	0.0280 (6)	0.0765 (10)	0.0478 (7)	−0.0013 (6)	−0.0061 (5)	0.0182 (6)
C1	0.0289 (7)	0.0296 (7)	0.0295 (7)	0.0032 (5)	0.0043 (5)	−0.0011 (5)
C2	0.0418 (8)	0.0309 (7)	0.0307 (7)	0.0054 (6)	0.0077 (6)	0.0029 (6)
C3	0.0416 (8)	0.0309 (7)	0.0356 (8)	−0.0024 (6)	0.0145 (6)	0.0004 (6)
C4	0.0303 (7)	0.0372 (8)	0.0402 (8)	−0.0051 (6)	0.0093 (6)	−0.0060 (6)
C5	0.0281 (7)	0.0369 (8)	0.0315 (7)	−0.0019 (6)	0.0028 (5)	−0.0040 (6)
C6	0.0261 (7)	0.0279 (7)	0.0264 (6)	0.0002 (5)	0.0044 (5)	−0.0029 (5)
C7	0.0256 (6)	0.0274 (7)	0.0251 (6)	0.0016 (5)	0.0007 (5)	−0.0012 (5)
C8	0.0263 (6)	0.0307 (7)	0.0256 (6)	−0.0004 (5)	0.0042 (5)	−0.0006 (5)
C9	0.0291 (7)	0.0273 (7)	0.0303 (7)	−0.0015 (5)	0.0017 (5)	−0.0004 (5)
C10	0.0367 (8)	0.0374 (8)	0.0327 (7)	−0.0132 (6)	0.0032 (6)	0.0016 (6)
C11	0.0377 (9)	0.0749 (14)	0.0572 (11)	0.0069 (9)	0.0193 (8)	0.0207 (10)
C12	0.0290 (7)	0.0491 (10)	0.0460 (9)	0.0061 (7)	0.0093 (6)	0.0064 (7)
C13	0.0251 (7)	0.0342 (7)	0.0332 (7)	−0.0023 (5)	0.0004 (5)	0.0018 (6)
C14	0.0261 (7)	0.0326 (7)	0.0269 (6)	0.0019 (5)	0.0023 (5)	0.0009 (5)
C15	0.0266 (7)	0.0431 (9)	0.0333 (7)	0.0032 (6)	0.0009 (6)	0.0046 (6)
C16	0.0270 (6)	0.0284 (7)	0.0267 (7)	−0.0024 (5)	0.0001 (5)	−0.0003 (5)
C17	0.0346 (8)	0.0390 (8)	0.0345 (8)	0.0058 (6)	0.0007 (6)	−0.0031 (6)
C18	0.0330 (8)	0.0438 (9)	0.0550 (10)	0.0062 (7)	−0.0019 (7)	0.0089 (8)
C19	0.0363 (8)	0.0534 (10)	0.0456 (9)	−0.0102 (7)	−0.0133 (7)	0.0196 (8)
C20	0.0456 (9)	0.0494 (10)	0.0288 (7)	−0.0187 (8)	−0.0053 (6)	0.0039 (7)
C21	0.0363 (8)	0.0328 (7)	0.0287 (7)	−0.0073 (6)	0.0031 (6)	−0.0022 (6)

### Geometric parameters (Å, °)

**Table:**

O1—C7	1.3491 (17)	C9—H9A	1.0000
O1—C8	1.4524 (16)	C10—C11	1.498 (3)
O2—C1	1.3751 (17)	C11—C12	1.521 (3)
O2—C15	1.3828 (18)	C11—H11A	0.9900
O3—C10	1.205 (2)	C11—H11B	0.9900
O4—C15	1.2112 (19)	C12—C13	1.543 (2)
C1—C2	1.389 (2)	C12—H12A	0.9900
C1—C6	1.394 (2)	C12—H12B	0.9900
C2—C3	1.380 (2)	C13—C14	1.503 (2)
C2—H2A	0.9500	C13—H13A	1.0000
C3—C4	1.394 (2)	C14—C15	1.446 (2)
C3—H3A	0.9500	C16—C17	1.385 (2)
C4—C5	1.384 (2)	C16—C21	1.386 (2)
C4—H4A	0.9500	C17—C18	1.386 (2)
C5—C6	1.3999 (19)	C17—H17A	0.9500
C5—H5A	0.9500	C18—C19	1.375 (3)
C6—C7	1.4483 (19)	C18—H18A	0.9500
C7—C14	1.3548 (19)	C19—C20	1.380 (3)
C8—C16	1.5050 (19)	C19—H19A	0.9500
C8—C9	1.5359 (19)	C20—C21	1.388 (2)
C8—H8A	1.0000	C20—H20A	0.9500
C9—C13	1.534 (2)	C21—H21A	0.9500
C9—C10	1.540 (2)		
			
C7—O1—C8	116.22 (10)	C12—C11—H11A	110.6
C1—O2—C15	121.99 (11)	C10—C11—H11B	110.6
O2—C1—C2	117.04 (13)	C12—C11—H11B	110.6
O2—C1—C6	121.37 (12)	H11A—C11—H11B	108.7
C2—C1—C6	121.59 (14)	C11—C12—C13	103.48 (14)
C3—C2—C1	118.94 (14)	C11—C12—H12A	111.1
C3—C2—H2A	120.5	C13—C12—H12A	111.1
C1—C2—H2A	120.5	C11—C12—H12B	111.1
C2—C3—C4	120.66 (14)	C13—C12—H12B	111.1
C2—C3—H3A	119.7	H12A—C12—H12B	109.0
C4—C3—H3A	119.7	C14—C13—C9	111.33 (12)
C5—C4—C3	120.06 (14)	C14—C13—C12	114.99 (13)
C5—C4—H4A	120.0	C9—C13—C12	104.62 (12)
C3—C4—H4A	120.0	C14—C13—H13A	108.6
C4—C5—C6	120.20 (14)	C9—C13—H13A	108.6
C4—C5—H5A	119.9	C12—C13—H13A	108.6
C6—C5—H5A	119.9	C7—C14—C15	119.90 (13)
C1—C6—C5	118.54 (13)	C7—C14—C13	122.11 (13)
C1—C6—C7	116.98 (12)	C15—C14—C13	117.96 (13)
C5—C6—C7	124.48 (13)	O4—C15—O2	116.59 (14)
O1—C7—C14	123.74 (13)	O4—C15—C14	125.49 (15)
O1—C7—C6	114.72 (12)	O2—C15—C14	117.93 (13)
C14—C7—C6	121.54 (13)	C17—C16—C21	119.32 (14)
O1—C8—C16	106.85 (11)	C17—C16—C8	120.64 (13)
O1—C8—C9	109.60 (11)	C21—C16—C8	120.00 (13)
C16—C8—C9	113.09 (12)	C16—C17—C18	120.36 (15)
O1—C8—H8A	109.1	C16—C17—H17A	119.8
C16—C8—H8A	109.1	C18—C17—H17A	119.8
C9—C8—H8A	109.1	C19—C18—C17	120.06 (17)
C13—C9—C8	110.72 (12)	C19—C18—H18A	120.0
C13—C9—C10	103.28 (12)	C17—C18—H18A	120.0
C8—C9—C10	110.45 (12)	C18—C19—C20	120.08 (15)
C13—C9—H9A	110.7	C18—C19—H19A	120.0
C8—C9—H9A	110.7	C20—C19—H19A	120.0
C10—C9—H9A	110.7	C19—C20—C21	120.03 (15)
O3—C10—C11	126.03 (15)	C19—C20—H20A	120.0
O3—C10—C9	124.73 (16)	C21—C20—H20A	120.0
C11—C10—C9	109.21 (13)	C16—C21—C20	120.14 (15)
C10—C11—C12	105.83 (13)	C16—C21—H21A	119.9
C10—C11—H11A	110.6	C20—C21—H21A	119.9
			
C15—O2—C1—C2	175.79 (14)	C8—C9—C13—C14	36.94 (16)
C15—O2—C1—C6	−3.8 (2)	C10—C9—C13—C14	155.18 (12)
O2—C1—C2—C3	−179.40 (13)	C8—C9—C13—C12	−87.86 (14)
C6—C1—C2—C3	0.2 (2)	C10—C9—C13—C12	30.38 (15)
C1—C2—C3—C4	0.2 (2)	C11—C12—C13—C14	−159.98 (15)
C2—C3—C4—C5	−0.4 (2)	C11—C12—C13—C9	−37.54 (17)
C3—C4—C5—C6	0.1 (2)	O1—C7—C14—C15	176.95 (13)
O2—C1—C6—C5	179.16 (13)	C6—C7—C14—C15	−3.5 (2)
C2—C1—C6—C5	−0.4 (2)	O1—C7—C14—C13	−5.1 (2)
O2—C1—C6—C7	−1.1 (2)	C6—C7—C14—C13	174.40 (13)
C2—C1—C6—C7	179.32 (13)	C9—C13—C14—C7	−6.2 (2)
C4—C5—C6—C1	0.3 (2)	C12—C13—C14—C7	112.55 (16)
C4—C5—C6—C7	−179.48 (14)	C9—C13—C14—C15	171.73 (13)
C8—O1—C7—C14	−17.6 (2)	C12—C13—C14—C15	−69.51 (18)
C8—O1—C7—C6	162.78 (12)	C1—O2—C15—O4	−175.20 (15)
C1—C6—C7—O1	−175.67 (12)	C1—O2—C15—C14	5.1 (2)
C5—C6—C7—O1	4.1 (2)	C7—C14—C15—O4	178.92 (16)
C1—C6—C7—C14	4.7 (2)	C13—C14—C15—O4	0.9 (3)
C5—C6—C7—C14	−175.52 (14)	C7—C14—C15—O2	−1.4 (2)
C7—O1—C8—C16	171.88 (11)	C13—C14—C15—O2	−179.35 (13)
C7—O1—C8—C9	49.00 (16)	O1—C8—C16—C17	−40.89 (18)
O1—C8—C9—C13	−58.49 (15)	C9—C8—C16—C17	79.79 (17)
C16—C8—C9—C13	−177.59 (11)	O1—C8—C16—C21	141.38 (13)
O1—C8—C9—C10	−172.27 (12)	C9—C8—C16—C21	−97.94 (15)
C16—C8—C9—C10	68.63 (15)	C21—C16—C17—C18	1.0 (2)
C13—C9—C10—O3	165.87 (15)	C8—C16—C17—C18	−176.74 (15)
C8—C9—C10—O3	−75.70 (19)	C16—C17—C18—C19	−0.8 (3)
C13—C9—C10—C11	−12.28 (18)	C17—C18—C19—C20	0.1 (3)
C8—C9—C10—C11	106.14 (16)	C18—C19—C20—C21	0.4 (3)
O3—C10—C11—C12	170.98 (17)	C17—C16—C21—C20	−0.5 (2)
C9—C10—C11—C12	−10.9 (2)	C8—C16—C21—C20	177.23 (13)
C10—C11—C12—C13	29.6 (2)	C19—C20—C21—C16	−0.2 (2)

### Hydrogen-bond geometry (Å, °)

**Table:**

*D*—H···*A*	*D*—H	H···*A*	*D*···*A*	*D*—H···*A*
C8—H8A···O3^i^	1.00	2.45	3.4042 (19)	160
C17—H17A···O2^ii^	0.95	2.54	3.322 (2)	140

Symmetry codes: (i) −*x*+1/2, *y*+1/2, −*z*+1/2; (ii) −*x*+1, −*y*, −*z*.
